# Determinants of poor sleep quality in adults during the coronavirus disease pandemic: *COVID-Inconfidentes*, a population-based study

**DOI:** 10.1590/1516-3180.2022.0139.R1.19082022

**Published:** 2022-10-28

**Authors:** Luiz Antônio Alves de Menezes, Luciano Garcia Lourenção, Amanda Cristina de Souza Andrade, Júlia Cristina Cardoso Carraro, George Luiz Lins Machado-Coelho, Adriana Lúcia Meireles

**Affiliations:** I MSc. Nutritionist and Doctoral Student, Postgraduate Program in Health and Nutrition, Universidade Federal de Ouro Preto (UFOP), Ouro Preto (MG), Brazil.; II MSc, PhD. Nurse and Associated Professor, Nursing School, Universidade Federal do Rio Grande (FURG), Rio Grande (RS), Brazil.; III MSc, PhD. Statistics and Associated Professor, Institute of Collective Health, Universidade Federal do Mato Grosso (UFMT), Cuiabá (MT), Brazil.; IV PhD. Nutritionist and Associated Professor, School of Nutrition, Universidade Federal de Ouro Preto (UFOP), Ouro Preto (MG), Brazil.; V MD, MSc, PhD. Epidemiologist and Associated Professor, School of Medicine, Universidade Federal de Ouro Preto (UFOP), Ouro Preto (MG), Brazil.; VI MSc, PhD. Nutritionist and Associated Professor, School of Nutrition, Universidade Federal de Ouro Preto (UFOP), Ouro Preto (MG), Brazil.

**Keywords:** Coronavirus infections, Sleep deprivation, Health surveys, Body weight changes, Sunlight, Anxiety, Coronavirus disease, Insufficient sleep, Anxiousness, Sleep latencies, Vitamin D deficiencies, Hygiene, Sleep

## Abstract

**BACKGROUND:**

The coronavirus disease (COVID-19) pandemic has adversely affected the health of the global population, with sleep quality being one of the affected parameters.

**OBJECTIVES:**

To evaluate sleep quality and its associated factors in adults during the COVID-19 pandemic in Brazil.

**DESIGN AND SETTING:**

A population-based cross-sectional serological survey of 1,762 adults in the Iron Quadrangle region of Brazil.

**METHODS:**

The Pittsburgh Sleep Quality Index was used to assess sleep quality. Sociodemographic variables, health conditions, health-related behaviors, anxiety, vitamin D levels, weight gain/loss, and pandemic characteristics were assessed using a structured questionnaire. Univariate and multivariate analyses using Poisson regression with robust variance were performed to identify factors associated with sleep quality.

**RESULTS:**

More than half of the participants reported poor sleep quality (52.5%). Multivariate analysis revealed that the factors associated with poor sleep quality included living alone (prevalence ratio [PR] = 1.34; 95% confidence interval [CI]: 1.04–1.73), anxiety disorder (PR = 1.32; 95% CI: 1.08–1.62), 5.0% weight loss (PR = 1.21; 95% CI: 1.02–1.44), 5.0% weight gain (PR = 1.27; 95% CI: 1.03–1.55), vitamin D deficiency (PR = 1.16; 95% CI: 1.01–1.35), and COVID-19 symptoms (PR = 1.29; 95% CI: 1.10–1.52).

**CONCLUSIONS:**

Our study revealed that more than half of the participants experienced poor sleep quality during the COVID-19 pandemic. Factors associated with poor sleep quality included vitamin D deficiency and weight changes related to the pandemic.

## INTRODUCTION

Sleep is essential for maintaining physiological parameters and plays an important role in hormone release and the regulation of cardiovascular activities and glucose levels.^1^ In addition, Poor sleep quality, particularly if chronic, may adversely affect the immune system components, disrupting antibody production after vaccination or previous contact with the viral agent. This could lead to increased vulnerability to infectious diseases such as coronavirus disease (COVID-19).^2^

From the beginning of the pandemic to almost two years later, Brazil has been one of the most affected countries. It remains in the top five countries with highest number of infected people and deaths due to COVID-19.^3^ Owing to the highly contagious nature of COVID-19 and limited knowledge regarding its natural history, several control measures have been adopted, such as practice of respiratory hygiene, use of masks, and implementation of social restrictions.^4^

These measures, along with the pandemic scenario, have led to drastic changes in people’s lifestyle, such as reduced physical activity, changes in food intake, reduced sun exposure,^4,5^ and other factors that directly affect sleep quality.^6,7^

## OBJECTIVE

As a pandemic tends to alter the daily routine and life habits of the population,^8^ this study aimed to evaluate sleep quality and its associated factors during the COVID-19 pandemic.

## METHODS

### Study design

This cross-sectional household population-based serological study is part of the COVID-*Inconfidentes* project (Epidemiological Surveillance of COVID-19 in the region of *Inconfidentes*, Minas Gerais). In this study, a seroepidemiological survey of 1,762 adults was conducted to determine the prevalence of COVID-19 and perform a situational assessment of the health-related aspects of this population. Data were collected on weekends between October and December 2020 in two medium-sized cities located in the central region of the state of Minas Gerais, known as the Iron Quadrangle. The Research Ethics Committee of the Federal University of Minas Gerais approved this project on September 22, 2020 (certificate of ethics submission: No. 32815620.0.1001.5149). All procedures adopted in this study were in accordance with the principles of the Declaration of Helsinki and the Brazilian guidelines and standards for human research. Written informed consent was obtained from all participants.

The survey was conducted in three stages at 21-day intervals, and different census sectors were evaluated in each city. The complex sample size calculation was based on the population estimate for each city, considering a confidence level of 95%, design effect of 1.5, and the parameters presented in a previous study.^9^

A three-stage conglomerate sampling design was adopted as follows: census sector (randomly selected for each stage and without replacement), households (selected by a systematic sampling process), and residents (one resident selected randomly). The sample weight of each selected unit (census tract, household, and individual) was calculated and adjusted to compensate for the loss of interviews owing to non-response, and the weights of the household and the selected resident were calibrated.^9^

### Data collection

The data collection process included listing and approaching households during weekends to enhance the participation of residents who worked during the week, thus increasing the representativeness of this population group.

Face-to-face interviews were conducted by trained interviewers, using a structured questionnaire to collect data on sociodemographic variables, health conditions, pandemic characteristics, and sleep quality. Sociodemographic variables included sex, age, marital and living status, education, family income, employment status (yes or no), and current work shift. Furthermore, we evaluated the work-from-home schedule. Health conditions included self-reported chronic diseases, divided into those with morbidity (reporting at least one disease) and without morbidity (no disease). Individuals were also assessed for chronic physical pain (physical pain present for ≥ 3 months), current smoking habit and alcohol consumption, and physical activity (grouped into: inactivity, at least 150–300 minutes of moderate-intensity aerobic physical activity per week, or at least 75–150 minutes of vigorous-intensity aerobic physical activity per week).^10^ Self-rated health was assessed as “very good,” “good,” “fair,” “poor,” and “very poor”.

Nutritional status was assessed based on body mass index (BMI). Self-reported weight and height were used to calculate BMI. Based on the BMI, the participants were classified as underweight (BMI < 18.5 kg/m^2^ if aged < 60 years; BMI < 23.0 kg/m^2^ if aged ≥ 60 years), eutrophic (BMI 18.5–24.9 kg/m^2^ if aged < 60 years; BMI 23.0–28.0 kg/m^2^ if aged ≥ 60 years), and overweight (BMI ≥ 25.0 kg/m^2^ if aged < 60 years; BMI ≥ 28.0 kg/m^2^ if aged ≥ 60 years).^11,12^ We also evaluated their weight change during the pandemic, according to the weight measured before and during the pandemic. To account for variability in weight change owing to differences in body mass, the percent change in total body weight from before the pandemic (March 2020) to the time of data collection (October to December 2020) was determined. A change in weight of ≥ 5% of body weight (gain or loss) was defined as a clinically significant change. Several studies have reported that a 5% gain in body weight has significant clinical effects not only on the risk of cardiovascular disease and diabetes mellitus^13,14^ but also on chronic pain,^15^ which is an important determinant of sleep quality.^16^ In addition, it has been recommended as a threshold for clinically relevant weight loss in several national and international guidelines.^17,18,19,20^

The average daily sun exposure was evaluated and classified as “insufficient” if exposure was < 30 minutes/day and “sufficient” if it was ≥ 30 minutes/day.^21^ We also evaluated a possible scenario of vitamin D deficiency, considering the extent of the time of sun exposure and consumption of food supplements fortified with vitamin D. Since there is no specific recommendation to determine sufficient vitamin D levels, we used the recommendations of Holick (2007) to classify the proposed components, which included an average sun exposure of 30 minutes or consumption of a supplement source of vitamin D (vitamin D sufficiency).^21^

Responses related to the COVID-19 pandemic were evaluated, such as presenting with at least one symptom in the last 15 days, social restriction since the beginning of the pandemic, any family member in the COVID-19 risk group, and the pandemic period. Furthermore, we asked about their daily routine activities during the pandemic.

### Measurement of sleep quality

The Pittsburgh Sleep Quality Index (PSQI) questionnaire was used to assess sleep quality. This instrument comprises 19 questions categorized into seven components: subjective sleep quality (C1), sleep latency (C2), sleep duration (C3), habitual sleep efficiency (C4), sleep disturbances (C5), use of sleep medication (C6), and daytime dysfunction (C7). The sum of the scores produces an overall score in the range of 0–21, with the highest score indicating the worst sleep quality. An overall score of > 5 indicates major difficulties in at least two components or moderate difficulties in more than three components.^22^ The Brazilian version of the PSQI has an overall reliability coefficient (Cronbach α) of 0.82, indicating a high degree of internal consistency.^23^

Herein, sleep quality was classified as good (PSQI score ≤ 5) or poor (PSQI score > 5). A PSQI score of ≥ 2 indicated moderate to severe difficulty in a sleep-specific domain (C1 to C7).^22^ This cut-off point was also used by Wang et al. in their study in 2020.^24^

### Statistical analysis

Statistical analyses were performed considering the complex design of the sample using the “svy” command of the Stata software (version 15.0; Stata Corp, College Station, Texas, United States). Data are presented as percentages and 95% confidence intervals (CI). Data were compared using the chi-square test and Bonferroni correction for multiple tests.^25^ Univariate and multivariate analyses were used to determine the association between sleep quality and sociodemographic factors, health conditions, and COVID-19-related variables. Data were analyzed using Poisson regression with robust variance^26^ to estimate the prevalence ratio (PR) and the respective 95% CI of the factors associated with sleep quality. Independent variables that had an association at a P value of 0.2 were used in multivariate regression with a stepwise backward elimination procedure controlling for the pandemic period variable. Collinearity among covariates was examined by calculating the variance inflation factor. The variables of anxiety and self-rated health were collinear and opted to retain anxiety disorders in the final model.

In addition, bivariate analysis was performed on the multivariate model of the interaction between the associated factors to verify a possible effect modification on sleep quality.

## RESULTS

### Characteristics and sleep quality of participants

Among the participants, women reported a high prevalence of abnormal PSQI scores in the subdomains of subjective sleep quality, sleep efficiency, and the use of sleep medications (P < 0.05). Furthermore, sleep medication use increased with increasing age, and daytime dysfunction was higher in the younger age group (P < 0.05) (Table 1). The mean PSQI score was 6.32 (95% CI: 6.03–6.62), and the prevalence of poor sleep quality was 52.5%. The highest prevalence rates for the abnormal specific sleep domains were for sleep latency (45.8%), sleep disturbance (36.8%), and sleep efficiency (20.1%) (see supplementary data: Figure S1 available in Google Drive: https://drive.google.com/file/d/1i0-Nvn6kRC4idX2rvWl0uDVWrSfwRKhI/view?usp=sharing).

**Table 1.** Distributions of abnormal Pittsburgh Sleep Quality Index subdomains by sleep quality, age and sex

	Abnormal Pittsburgh Sleep Quality Index subdomains^a^, n (%)						
	Subjective sleep quality	Sleep latency	Sleep duration	Sleep efficiency	Sleep disturbance	Use of sleep medications	Daytime dysfunction
**Total sample**	18.3 (14.9–22.4)	45.8 (41.6–50.1)	15.7 (12.3–19.8)	20.1 (16.7–24.1)	36.8 (32.0–41.9)	9.6 (7.6–12.1)	13.9 (11.0–17.5)
**Sex**							
Male	36.6 (27.2–47.2)	43.8 (34.6–53.5)	44.3 (35.2–53.7)	37.5 (28.7–47.1)	42.8 (32.9–53.2)	29.0 (19.9–40.3)	39.8 (27.7–53.3)
Female	63.4 (52.8–72.8)	56.2 (46.5–65.4)	55.7 (46.3–64.8)	62.5 (52.9–71.3)	57.2 (46.8–67.1)	71.0 (59.7–80.1)	60.2 (46.7–72.3)
P value	**0.043**	0.108	0.448	**0.032**	0.070	**0.001**	0.222
**Age**							
18–34 years	35.6 (25.6–47.7)	36.0 (29.0–43.6)	33.7 (22.0–47.8)	31.7 (22.4–42.8)	28.8 (21.0–38.1)	11.1 (5.6–20.9)	50.2 (38.0–62.3)
35–59 years	47.0 (35.3–59.0)	45.3 (37.2–53.7)	44.8 (32.4–57.8)	44.8 (36.8–53.0)	48.2 (37.7–58.9)	54.4 (43.2–65.1)	35.0 (25.2–46.3)
≥ 60 years	17.1 (12.4–23.1)	18.7 (14.9–23.0)	21.5 (15.2–29.6)	23.5 (18.1–30.0)	23.0 (17.0–30.2)	34.5 (24.8–45.5)	14.8 (9.7–21.9)
P value	0.854	0.971	0.746	0.385	0.157	**< 0.001**	**0.004**

Score for each domain ranges from 0 to 3 (no difficulty to severe difficulty), and a domain score ≥ 2 indicates abnormal sleep in the domain.

Among the participants, 51.9% were women, and the most prevalent age group was 35–59 years (45.6%). Most participants were married (53.2%), had > 9 years of schooling (68.8%), and had a family income ≤ 2 times the minimum wage (41.1%) (Table 2). More than half of the participants had at least one chronic disease (52.3%), consumed alcoholic beverages (58.2%), were physically inactive (69.2%), and were overweight (61.4%) (Table 3). At least 12% of the participants experienced 5.0% weight loss or gain during the pandemic (12.4% and 17.7%, respectively), 35.0% had a daily sun exposure of < 30 minutes, and 27.1% had vitamin D deficiency (Table 4).

**Table 2.** Sociodemographic characteristics according to sleep quality during pandemic

Characteristics	Total % (95% CI)	Sleep quality		*Prevalence ratio* (95% CI)	P*
		Good (PSQI ≤ 5) % (95% CI)	Poor (PSQI > 5) % (95% CI)		
Total		47.5 (43.6–51.4)	52.5 (48.6–56.4)	–	–
Sociodemographic					
**Sex**					
Male	48.1 (41.0–55.2)	47.2 (39.3–55.2)	43.8 (36.2–51.7)	1.00	
Female	51.9 (44.7–59.0)	52.8 (44.8–60.7)	56.2 (48.3–63.8)	*1.20 (1.05*–*1.36)*	*0.006*
**Age**					
18–34 years	35.6 (31.1–40.3)	38.7 (30.5–47.6)	32.8 (26.4–39.9)	1.00	
35–59 years	45.6 (41.1–50.2)	44.5 (37.0–52.3)	46.6 (38.8–54.5)	*1.11 (0.82*–*1.49)*	*0.496*
≥ 60 years	18.8 (15.5–22.7)	16.8 (12.4–22.3)	20.6 (16.4–25.7)	*1.22 (0.94*–*1.58)*	*0.122*
**Marital status**					
Married	53.2 (47.2–59.2)	58.0 (51.2–64.6)	48.9 (40.8–57.1)	1.00	
Unmarried	46.8 (40.8–52.8)	42.0 (35.4–48.8)	51.1 (42.8–59.2)	*1.17 (0.97*–*1.40)*	*0.091*
**Living status**					
Non-alone	95.3 (93.5–96.6)	97.5 (96.4–98.2)	99.3 (90.1–95.5)	1.00	
Alone	4.7 (3.4–6.5)	2.5 (1.7–3.6)	6.7 (4.5–9.9)	*1.44 (1.24*–*1.68)*	*< 0.001*
**Education**					
> 9 years	68.8 (64.0–73.3)	75.7 (69.1–81.2)	62.6 (54.5–70.1)	1.00	
≤ 9 years	31.2 (26.7–36.0)	24.3 (18.8–30.9)	37.4 (29.9–45.5)	*1.33 (1.09*–*1.64)*	*0.006*
**Family Income**					
≤ 2 MW	41.1 (35.6–46.8)	38.9 (30.4–48.0)	43.0 (34.0–52.5)	1.00	
> 2 to ≤ 4 MW	32.0 (26.9–37.5)	31.4 (24.6–39.1)	32.5 (26.2–39.5)	*0.99 (0.76*–*1.29)*	*0.955*
> 4 MW	26.9 (22.0–32.5)	29.7 (21.4–39.7)	24.5 (18.3–31.9)	*0.86 (0.60*–*1.24)*	*0.428*
**Workers**					
No	47.5 (42.7–52.3)	44.2 (36.5–52.2)	50.5 (43.2–57.8)	1.00	
Yes	52.5 (47.7–57.3)	55.8 (47.8–63.5)	49.5 (42.2–56.8)	*0.89 (0.71*–*1.11)*	*0.299*
**Work from home** ^**a**^					
No	61.4 (53.5–68.8)	57.6 (57.4–76.3)	55.0 (44.9–64.7)	1.00	
Yes	38.6 (31.2–46.5)	32.4 (23.7–42.6)	45.0 (35.3–55.1)	*1.30 (0.99*–*1.71)*	*0.056*
**Shift work**					
No	91.4 (86.3–94.7)	89.4 (78.1–95.3)	93.2 (88.6–96.1)	1.00	
Yes	8.6 (5.3–13.7)	10.6 (4.7–21.9)	6.8 (3.9–11.4)	*0.77 (0.41*–*1.43)*	*0.405*

PSQI = Pittsburgh Sleep Quality Index; MW = Minimum wage; CI = confidence interval. ^a^Percentage of active workers who were working at home. *Prevalence ratio estimated by Poisson regression with robust variance.* *In order to avoid the type 1 error, the Bonferroni correction for multiple [9] tests, was set at 0.005.

**Table 3.** Health conditions according to sleep quality during pandemic

Characteristics	Total	Sleep quality		***Prevalence ratio*** (95% CI)	P*
		Good (PSQI ≤ 5) % (95% CI)	Poor (PSQI > 5) % (95% CI)		
Health conditions					
**Chronic diseases**					
No	47.7 (41.3–54.2)	52.9 (44.1–61.5)	43.0 (35.8–50.5)	1.00	
Yes	52.3 (45.8–58.7)	47.1 (38.5–55.9)	57.0 (49.4–64.2)	*1.22 (1.10*–*1.35)*	*< 0.001*
**Chronic pain**					
No	65.7 (61.4–69.7)	75.0 (66.9–81.7)	57.3 (49.5–64.7)	1.00	
Yes	34.3 (30.3–38.6)	25.0 (18.3–33.1)	42.7 (35.3–50.5)	*1.44 (1.13*–*1.83)*	*0.004*
**Healthcare** ^**a**^					
Anxiety disorder	20.6 (17.0–24.8)	12.8 (7.6–20.8)	27.7 (22.9–33.1)	*1.49 (1.22*–*1.83)*	*< 0.001*
Depression	12.7 (9.6-16.6)	7.0 (3.0–15.8)	17.9 (14.1–22.4)	*1.52 (1.17*–*1.97)*	*0.002*
**Self-rated health**					
Good	77.4 (73.3–80.9)	87.5 (83.9–90.3)	68.2 (61.7–74.1)	1.00	
Poor	22.6 (19.0–26.7)	12.5 (9.7–16.1)	31.8 (25.9–38.3)	*1.62 (1.42*–*1.83)*	*< 0.001*
**Behaviors**					
Current smoking	17.0 (13.3–21.4)	18.3 (12.6–25.9)	15.8 (11.3–21.5)	*0.90 (0.66*–*1.22)*	*0.508*
Current alcohol consumption	58.2 (52.1–64.0)	62.2 (55.9–68.2)	54.5 (46.8–62.1)	*1.15 (0.89*–*1.33)*	*0.053*
**Physical activity**					
Physically active	30.8 (26.2–35.8)	35.1 (28.2–42.5)	26.9 (21.2–33.5)	1.00	
Physically inactive	69.2 (64.2–73.7)	64.9 (57.5–71.8)	73.1 (66.5–78.8)	*1.22 (0.96*–*1.55)*	*0.096*
**Nutritional status**					
Eutrophic	36.0 (30.7–41.7)	34.5 (27.7–42.1)	37.4 (29.8–45.7)	1.00	
Underweight	2.6 (1.8–3.6)	2.4 (1.5–3.8)	2.7 (1.6–4.4)	*1.02 (0.73*–*1.43)*	*0.918*
Overweight	61.4 (55.6–66.9)	63.0 (55.4–70.0)	59.9 (51.6–67.7)	*0.95 (0.76*–*1.19)*	*0.676*

PSQI = Pittsburgh Sleep Quality Index; CI = confidence interval. ^a^Anxiety disorder and depression (evaluated by self-report of medical diagnosis). *Prevalence ratio estimated by Poisson regression with robust variance*. *In order to avoid the type 1 error, the Bonferroni correction for multiple [7] tests, was set at 0.007.

**Table 4.** Coronavirus disease 2019 (COVID-19) related variables according to sleep quality during pandemic

Characteristics	Total	Sleep quality		***Prevalence ratio*** (95% CI)	P*
		Good (PSQI ≤ 5) % (95% CI)	Poor (PSQI > 5) % (95% CI)		
**Weight change** ^**a**^					
Δ -5% to +5%	69.9 (64.8–74.5)	76.6 (71.6–80.9)	63.9 (56.5–70.8)	1.00	
Δ ≤ -5%	12.4 (9.3–16.4)	10.1 (7.3–13.7)	14.5 (9.7–21.0)	*1.27 (1.04*–*1.55)*	*0.022*
Δ ≥ +5%	17.7 (14.8–21.1)	13.3 (9.9–17.8)	21.6 (16.7–27.5)	*1.32 (1.09*–*1.59)*	*0.004*
**Exposure sun**					
≥ 30 minutes/day	64.5 (59.3–70.3)	69.1 (62.6–74.8)	61.3 (53.7–68.4)	1.00	
< 30 minutes/day	35.0 (29.7–40.7)	30.9 (25.1–37.4)	38.7 (31.6–46.3)	*1.16 (0.98*–*1.35)*	*0.074*
**Vitamin D supplementation**					
No	77.9 (73.3–81.9)	80.2 (74.6–84.8)	75.9 (69.4–81.4)	1.00	
Yes	22.1 (18.0–26.7)	19.8 (15.2–25.4)	24.1 (18.6–30.6)	*1.14 (0.93*–*1.40)*	*0.212*
**Vitamin D scenario** ^**b**^					
Sufficient	72.9 (68.1–77.3)	76.7 (72.3–81.1)	69.3 (62.3–75.4)	1.00	
Insufficient	27.1 (22.7–31.9)	23.0 (18.9–27.7)	30.7 (24.5–37.7)	*1.19 (1.03*–*1.37)*	*0.020*
**SARS-CoV-2**					
Seronegative	94.8 (93.0–96.2)	94.9 (91.8–96.9)	94.7 (92.1–96.5)	1.00	
Seropositive	5.2 (3.8–7.0)	5.1 (3.1–8.2)	5.3 (3.5–7.9)	*1.12 (0.79*–*1.59)*	*0.518*
**Symptoms of COVID-19**					
No	71.4 (66.7–75.8)	79.8 (75.0–83.8)	63.8 (56.7–70.4)	1.00	
Yes	28.6 (24.2–33.3)	20.2 (16.2–25.0)	36.2 (29.6–43.3)	*1.44 (1.24*–*1.65)*	*< 0.001*
**Risk group in family**					
No	40.8 (33.8–48.2)	46.2 (38.8–53.7)	36.0 (28.2–44.5)	1.00	
Yes	59.2 (51.8–66.2)	53.8 (46.3–61.2)	64.0 (55.5–71.8)	*1.23 (1.07*–*1.42)*	*0.003*
**Pandemic period**					
8.5–9 months	18.9 (14.6–24.1)	22.5 (16.6–29.6)	15.6 (11.7–20.6)	1.00	
7–8.4 months	81.1 (75.9–85.4)	77.5 (70.4–83.3)	84.4 (79.4–88.3)	*1.26 (1.04*–*1.53)*	*0.018*
**Daily routine in pandemi**c					
Social contact restriction	62.6 (58.3–66.7)	56.7 (48.3–64.7)	68.0 (61.3–73.9)	*1.28 (0.98*–*1.66)*	*0.069*
Physical activity in the street	23.9 (20.3–28.0)	26.1 (18.8–34.9)	22.0 (18.1–26.5)	*0.90 (0.68*–*1.18)*	*0.428*
Physical activity in the gym	10.2 (6.7–15.2)	14.2 (8.5–22.6)	6.6 (3.7–11.4)	*0.62 (0.37*–*1.01)*	*0.058*

PSQI = Pittsburgh Sleep Quality Index; SARS-CoV-2 = severe acute respiratory syndrome coronavirus 2. ^a^Weight change during the pandemic (self-reported weight). ^b^Sufficient: Sun exposure > 30 minutes/day or vitamin D supplements; Insufficient: Sun exposure < 30 minutes/day and no vitamin D supplements. *Prevalence ratio estimated by Poisson regression with robust variance.* *In order to avoid the type 1 error, the Bonferroni correction for multiple [9] tests, was set at 0.005.

### Factors associated with poor sleep quality

In the multivariate model, the following factors were significantly associated with poor sleep quality: living alone (PR = 1.34; 95% CI: 1.04–1.73), anxiety disorder (PR = 1.32; 95% CI: 1.08–1.62), 5.0% weight loss (PR = 1.21; 95% CI: 1.02–1.44), 5.0% weight gain (PR = 1.27; 95% CI: 1.03–1.55), vitamin D deficiency (PR = 1.16; 95% CI: 1.01–1.35), and COVID-19 symptoms (PR = 1.29; 95% CI: 1.10–1.52).

**Figure 1:**
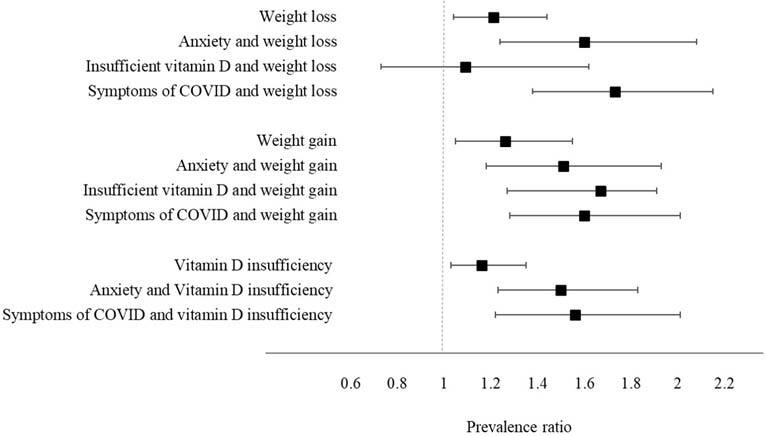
Bivariate association adjusted for weight change, and vitamin D scenario insufficiency with individual parameters associated with poor sleep quality during the COVID-19 pandemic.

Based on the factors associated with sleep quality obtained in the aforementioned adjusted model (Table 5), a chance modification analysis for poor sleep quality was performed, assuming the presence of combined changes in these variables (Figure 1). Overall, we observed that the assessed variables had a gradient of probability for sleep quality, with the PR of poor sleep quality increasing when two concurrently altered variables were analyzed. The worst scenarios were the concurrence of COVID-19 symptoms and weight loss (PR = 1.72; 95% CI: 1.38–2.15) and vitamin D deficiency and weight gain (PR = 1.67; 95% CI: 1.19–1.91). Only weight loss when evaluated concomitantly with vitamin D deficiency was not significant (PR = 1.09; 95% CI: 0.73–1.62).

**Table 5.** Multivariate analysis of factors associated with poor sleep quality

Variables	Univariate analysis			Multivariate analysis		
	PR	95% CI	P	PR	95% CI	P
**Living status**						
Non-alone	1.00	–		1.00	–	
Alone	*1.44*	*1.24*–*1.68*	***< 0.001***	*1.34*	*1.04*–*1.73*	***0.026***
**Anxiety disorder^a^**						
No	1.00	–		1.00	–	
Yes	*1.49*	*1.22*–*1.83*	***< 0.001***	*1.32*	*1.08*–*1.62*	***0.008***
**Weight change^b^**						
Δ -5% to +5%	1.00	–		1.00	–	
Δ ≤ -5%	*1.27*	*1.04*–*1.55*	***0.022***	*1.21*	*1.02*–*1.44*	***0.028***
Δ ≥ +5%	*1.32*	*1.09*–*1.59*	***0.004***	*1.27*	*1.03*–*1.55*	***0.026***
**Vitamin D scenario^c^**						
Sufficient	1.00	–		1.00	–	
Insufficient	*1.19*	*1.03*–*1.37*	***0.020***	*1.16*	*1.01*–*1.35*	***0.043***
**Symptoms of COVID-19**						
No	1.00	–		1.00	–	
Yes	*1.43*	*1.24*–*1.65*	***< 0.001***	*1.29*	*1.10*–*1.52*	***0.003***

Multivariate model adjusted for the best fit model, by the technique stepwise backward. Model included sex, age, living status, anxiety, weight change, vitamin D scenario, symptoms of coronavirus disease (COVID) and pandemic period. ^a^ Anxiety disorder and depression (evaluated by self-report of medical diagnosis). ^b^ Weight change during the pandemic (self-reported weight). ^c^ Sufficient: Sun exposure > 30 min/day or vitamin D supplements; Insufficient: Sun exposure < 30 minutes/day and no vitamin D supplements. CI = confidence interval; PR = prevalence ratio; COVID-19 = coronavirus disease 2019. *Prevalence ratio estimated by Poisson regression with robust variance.*

Weight loss is defined as a loss of 5% or more during the pandemic. Weight gain is defined as a gain of 5% or more during the pandemic (self-reported weight).

## DISCUSSION

This study investigated the prevalence of poor sleep quality and its associated factors during the COVID-19 pandemic. More than half of the population had poor sleep quality. The PR of poor sleep quality was higher in individuals living alone, with anxiety disorders, experiencing weight change during the pandemic, with vitamin D deficiency, and with COVID-19 symptoms. The most affected PSQI sub-domains were sleep latency, sleep disturbance, and sleep efficiency.

During the pandemic, several factors may have contributed to the alteration of normal sleep architecture. Hence, population studies are important because they allow us to evaluate how health outcomes affect people’s lives. However, only a few studies with this methodology using the PSQI have been conducted during the pandemic, which makes it difficult to compare the results. Our study, conducted from October to December 2020, reported a higher prevalence of poor sleep quality than studies conducted at the beginning of the pandemic, such as the systematic review by Krishnamoorthy et al. (2020), wherein, approximately 36% of the general population and 43% of healthcare workers, which were one of the most affected groups during the pandemic, reported poor sleep quality.^27^

Furthermore, a multicenter online survey conducted from April to May 2020 corroborates our results. In that survey, 5,056 individuals from Europe, North Africa, West Asia, and the Americas were evaluated, and a 52.0% prevalence of poor sleep quality was assessed using the PSQI.^28^ In Brazil, a study on 45,161 individuals from April to May 2020 showed that during the pandemic, 66.1% reported usual sleep problems. This was particularly noted in women aged 40–50 years, unemployed and physically inactive individuals, and those with a greater number of health problems.^29^ However, it should be noted that this study was conducted online, which would usually represent a more educated and higher-income group of the population and hence is different from a household survey.

During the pandemic, online tasks made the workday endless and affected sleep quality. Such a work schedule also reduced individuals’ sun exposure, as most people spending more time doing online tasks no longer commuted to work or lunch. Sun exposure is an important factor because it is the main source of endogenous vitamin D.^21^ We found that individuals with insufficient vitamin D levels had a higher PR for poor sleep quality than those with sufficient levels. This association may be explained by the intracellular distribution of vitamin D receptors in brain areas that regulate the sleep-wake cycle or through pro-inflammatory mediators. Vitamin D is also involved in the production of melatonin, an essential hormone in the regulation of circadian rhythm and sleep. Melatonin synthesis is controlled by the active form of vitamin D, 1,25(OH)_2_D, that induces the expression of tryptophan hydroxylase (the initial enzyme in the melatonin synthesis pathway).^30^ This suggests a possible role for vitamin D deficiency in sleep disturbances.^31,32^ These results were found in a previous study on mining workers conducted in the same region as that of our study. When evaluating sleep quality using polysomnography, the gold standard method, workers with hypovitaminosis D had more sleep disturbances than those without it.^33^ The routine of these workers was similar to that of people confined during the COVID-19 pandemic, since they were off-road machinery drivers who spent most of their time on machines without access to sunlight.^34^

An additional variable associated with poor sleep quality in our study was weight change during the pandemic. Individuals who reduced or gained up to 5.0% of their body weight during the pandemic had a greater PR for poor sleep quality than those who did not experience weight change. Weight loss, when intentional, particularly in obese individuals, can be beneficial in improving sleep quality.^35^ However, unintentional weight loss may be related to increased physical and emotional stress or an imbalance between food supply and demand. A systematic review conducted between July 2020 and February 2021 found that during the pandemic, 11.1–32.0% of the total 469,362 participants had experienced weight loss.^36^ For some people, the lockdown provided more time to cook and eat better; however, most people developed malnutrition and experienced weight loss owing to inflated food prices and food insecurity. In Brazil, more than half of the households (59.4%) experienced food insecurity during the pandemic.^37^ Insufficient food consumption of adequate quantity and quality can have severe health effects, such as poor mental health and increased likelihood of diseases,^37^ increasing the chances of poor sleep quality and vulnerability to COVID-19.

In addition, pandemic confinement was associated with weight gain in 7.2–72.4% of participants in a previous systematic review.^36^ Excess weight interferes with sleep quality in several aspects, including anatomical factors such as airway obstruction or inflammatory factors such as increased cytokines, which can induce sleep disturbances by altering the sleep-wake rhythm.^38^ Furthermore, there is a strong association between poor sleep quality and the risk of obesity, as demonstrated in previous longitudinal studies. In a cohort of 83,377 Americans, comprising non-obese men and women at baseline, participants reporting < 5 hours of sleep per night had an approximately 40% higher risk of developing obesity than those reporting 7–8 hours of sleep (for men, odds ratio [OR] = 1.45, 95% CI: 1.06–1.99; for women, OR = 1.37, 95% CI: 1.04–1.79).^39^ Furthermore, a recent study evaluating sleep disturbances in 4,384 health professionals during COVID-19 found that weight loss or weight gain were independent predictors of new-onset or worsening of preexisting insomnia (for weight loss, OR = 1,772, 95% CI: 1,453–2,161; for weight gain, OR = 1,468; 95% CI: 1,249–1,728).^40^

Unfortunately, the fear and uncertainty caused by the pandemic and threat to survival, among other factors, are some of the main problems encountered during the pandemic that have greatly influenced the quality of life and mental health.^4^ Of all the factors evaluated in our study, anxiety and living alone were the most strongly associated with poor sleep quality.

Pandemic conditions and social isolation affect many aspects of living conditions and the health status of the population, particularly mental health. In Brazil, 52.6% of the population reported frequently feeling anxious or nervous.^6^ Anxiety, especially generalized anxiety disorder, has been described as one of the most important consequences of sleep deprivation.^41^ A study conducted during the initial weeks of the lockdown in Italy showed that reduced sleep quality was directly related to the days spent at home in confinement, as mental health plays an important role in mediating sleep quality.^42^ A systematic review and meta-analysis of 345,270 participants from 39 countries showed consistent results regarding the association between sleep quality and psychological distress. The corrected pooled estimated prevalence of sleep problems was 18% in the general population and was positively associated with anxiety (Fisher z-score = 0.48; 95% CI: 0.41–0.54).^41^

The psychological impact during a pandemic is common and expected, as demonstrated by Brooks et al. (2020), who studied previous epidemics. The main psychological stressors were duration of quarantine, fear of infection, feelings of frustration and annoyance, inadequate information about disease precautions, unemployment, financial losses, and stigma associated with the disease.^4^

In addition to these factors, we also found that participants who experienced co-occurrence of two associated factors had a higher PR for poor sleep quality than those who did not. These results are important because the social and health effects of the pandemic have rendered many individuals vulnerable to the co-occurrence of factors that negatively interfere with sleep quality. In this context, vitamin D deficiency and weight gain are closely related factors that can occur simultaneously.^21,31^ Therefore, the co-occurrence of these factors can increase the PR of poor sleep quality, as shown in this study. To the best of our knowledge, this is the first study to evaluate the co-occurrence of the factors associated with poor sleep quality during the COVID-19 pandemic.

Insufficient sleep directly affects the immune system and increases the risk for illness. Thus, we found a high prevalence of poor sleep quality during the COVID-19 pandemic, with several associated factors. Sleep quality may have been influenced by the COVID-19 pandemic and the government’s actions taken to contain it. Brazil is one of the countries with the highest number of deaths and the lowest percentage of vaccinated individuals.

Adequate sleep quality is an important factor to consider in a pandemic, given its beneficial effect on numerous health conditions and improvement of the immune response against opportunistic infections.^1^ Thus, a health-related emergency, such as the one we are currently experiencing, should be accompanied by adequate social support programs to mitigate the psychological, social, and economic effects and promote better circumstances to face such troubled times.

This study identified the important factors related to sleep quality during the pandemic; however, these findings should be interpreted with caution. In our study, causal relationships could not be determined because of the absence of previously available information on sleep quality. Furthermore, the variables were obtained by self-reporting, which may have caused underestimation of risk or overestimation of protective behaviors owing to differences in each individual’s perception of the pandemic and associated factors. However, the assessment of sleep quality needs to be performed subjectively since it considers the factors intrinsic to individuals’ perception of their sleep. Self-reported weight and height may have influenced these results; however, there are studies involving similar populations and strong methodological rigor that demonstrated high agreement with the measured values.^43,44^ Therefore, BMI computed from self-reported weight and height can be considered a valid measure in men and women of different sociodemographic groups.^43,44^ The strengths of this study include a representative random sample of the resident population from different socioeconomic strata, evaluation using a household survey, and face-to-face interviews during the COVID-19 pandemic, which increased the robustness of the study.

## CONCLUSION

Our study revealed that more than half of the participants had poor sleep quality during the COVID-19 pandemic. Moreover, factors associated with poor sleep quality were related to the pandemic, such as vitamin D deficiency and weight change. Therefore, future longitudinal and randomized intervention trials should be conducted to confirm the relevant associations. Thus, governing and regulatory bodies must provide subsidies for decision-making in chaotic socio-sanitary and epidemiological conditions to reduce the worsening of health conditions.
